# Simple but complex: aged care healthcare professionals’ perspectives on the design of a digital falls dashboard

**DOI:** 10.1186/s12911-025-03135-z

**Published:** 2025-09-29

**Authors:** Laura Dodds, Isabelle Meulenbroeks, S. Sandun Malpriya Silva, Kristiana Ludlow, Crisostomo Mercado, Karla Seaman, Nasir Wabe, Melissa Baysari, Johanna I. Westbrook, Amy D. Nguyen

**Affiliations:** 1 Centre for Health Systems and Safety Research, Australian Institute of Health Innovation, Macquarie University, Sydney, NSW 2109 Australia; 2 The MARCS Institute for Brain, Behaviour and Development, Western Sydney University, Westmead, NSW Australia; 3 Centre for Health Services Research, The University of Queensland, Brisbane, QLD Australia; 4 Susan Wakil School of Nursing and Midwifery, Faculty of Medicine and Health, The University of Sydney, Sydney, NSW Australia; 5 St Vincent’s Clinical Campus, UNSW Medicine, UNSW Sydney, Sydney, NSW Australia

**Keywords:** Aged care, Residential aged care facility, Nursing home, Digital health, Electronic health record, Clinical dashboard, Dashboard design

## Abstract

**Background:**

Digital dashboards are widely employed across healthcare settings to present data, supporting timely risk identification and enhancing clinical decision-making. Incorporating feedback from end-users into dashboard design supports their uptake and utilisation. The current study aimed to: (a) understand how healthcare professionals working in residential aged care gather, interpret, transfer and communicate clinical information especially for falls management; and (b) utilise co-design methods to determine healthcare professionals’ preferences for presentation, content and functionality of a digital falls dashboard to support delivery of care in residential aged care.

**Methods:**

Participants were recruited via aged care provider and primary health network contacts. Individual interviews with general practitioners (GPs) (*n* = 3) explored end-user needs including information needs for falls management, decision-making processes, and dashboard preferences. Dashboard prototypes were developed using the interview findings and published guidelines. Prototypes were then presented for feedback in eight workshops (*n* = 20 participants; residential aged care staff, GPs, and geriatricians) completed via videoconferencing or in-person to gain feedback. Interview and workshop transcripts were analysed using template analysis.

**Results:**

During interviews, GPs discussed difficulties in accessing aged care resident information, clinical decision-making in residential aged care, and use of decision support. During workshops, healthcare professionals shared feedback on the design, content, and functionality of dashboard prototypes. Healthcare professionals also discussed themes of human-technology interaction. This included mistrust of new digital tools and barriers to their use in residential aged care. The current study found that healthcare professionals want a dashboard that displays relevant resident data, such as medications, includes features for benchmarking, and provides detailed insights to support decision making. They expressed a need for evidence-based decision support but advocated for minimal alerts.

**Conclusions:**

Healthcare professionals were receptive to using a dashboard in residential aged care to minimise resident falls. They shared their design ideas in co-design interviews and workshops for a prospective dashboard. Findings informed the initial development and subsequent revisions of the dashboard to align with end-user preferences.

**Supplementary Information:**

The online version contains supplementary material available at 10.1186/s12911-025-03135-z.

## Introduction

General practitioners (GPs) collect important health information about residents who live in residential aged care facilities (RACFs; also known as nursing homes, care homes and long-term care). However, little is known about the information needs of GPs, as well as other healthcare professionals such as geriatricians and aged care nurses. Limited literature exists regarding how these healthcare professionals access information within RACFs [1]. Further, the process by which healthcare providers transfer resident information between settings of care can be different for each healthcare provider, and can involve paper-based, electronic or hybrid systems. There are currently limited systems for them to use to reconcile resident information across settings [2, 3]. 

Digital dashboards are interfaces which use visual analytics to display key information. Dashboards are becoming increasingly common in healthcare settings as a means of improving care quality. Dashboards support healthcare professionals to provide timely and evidence-based care through advanced features such as decision support functions and predictive analytics [4]. Uptake of dashboards in aged care has been limited by barriers such as immature information technology infrastructure, fragmented systems, resistance to change, limited digital literacy, lack of training and low resources [5, 6]. 

Falls are a critical outcome of interest in Australian RACFs. As part of the Australian National Aged Care Mandatory Quality Indicator Program (QI program with 11 indicators), aged care providers are required to report quarterly on the number of falls as well as the injurious falls and hospitalisations resulting from falls [7]. Each year, around 30–50% of Australian residents in RACFs experience falls, and40% of them have recurrent fall incidents [8]. Negative fall-related health outcomes occur frequently, including premature death and poor quality of life [9]. Fall-related care and outcomes incur considerable costs in Australia (> AU$498.2 million, annually) [10]. Falls in RACFs may be preventable through risk mitigation strategies and fall prevention interventions such as medication reconciliation, vitamin D prescription and exercise [11]. Thus, a dashboard that focuses on falls management in RACFs would be beneficial as it would allow timely identification of those residents who are at risk of having a fall, and alert healthcare professionals to deliver decision support to mitigate these risks.

The current study has been conducted as part of a larger group of studies to co-design, develop and evaluate a digital clinical falls dashboard for use in RACFs [4, 12–14]. To ensure effective care and widespread adoption, dashboards need to fit the needs and workflows of their end-users. Co-design, as proposed in the current study, will ensure the dashboard is outcomes-focused to support effective care and adaptability to support widespread adoption [14]. The falls dashboard will have information and decision support that aligns with current falls protocols, is highly specific to the needs of aged care residents and is effectively facilitated across RACFs. To create the co-designed dashboard, the current study aimed to: (a) understand how healthcare professionals working in residential aged care gather, interpret, transfer and communicate clinical information; and (b) utilise co-design methods to determine healthcare professionals’ preferences for presentation, content and functionality of a digital falls dashboard to support delivery of care in RACFs.

## Methods

### Study design

**Fig. 1:**
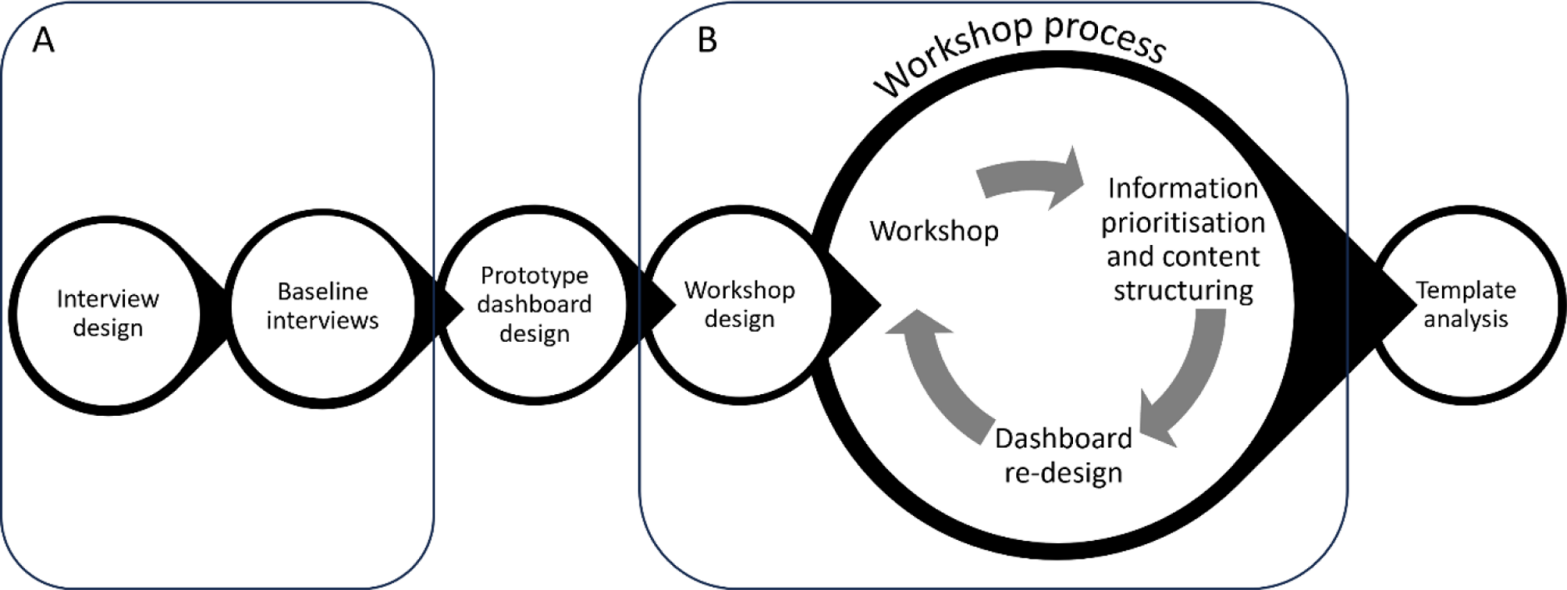
Tool development, data collection and analysis process for Stage 1 (**A**) and Stage 2 (**B**)

This study employed a co-design approach, across a 2-stage process (Fig. 1). In Stage 1, baseline interviews were conducted to understand how dashboard end-users currently use healthcare data and information, and to identify information requirements for a clinical dashboard for better decision-making in falls management (Fig. 1, A). In this research, “end user” refers to healthcare professionals: GPs, geriatricians and RACF staff including care workers and nurses. Findings from Stage 1 informed the design of dashboard prototypes which were presented in Stage 2. In Stage 2, participants engaged in rounds of iterative feedback, followed by rapid dashboard re-design in workshops (Fig. 1, B).

### Participants and setting

The wider research project consists of a partnership with one of the largest aged care providers in New South Wales, Australia. Participants were from the aged care partner facilities or were employed in the local primary health network. The local primary health network is an independent organisation that manages the coordination of health services and staff across regions in Australia. Eligibility criteria to participate in interviews or workshops included: (i) GPs, geriatricians or aged care staff currently working in RACFs, (ii) capacity to understand and communicate in English and (iii) the ability to provide informed consent.

### Recruitment

Recruitment occurred simultaneously to data collection at two time points. Stage 1 was conducted from September 2020-March 2021 and Stage 2 occurred from March-August 2022. Participants were recruited via emails, expression of interest flyers, and snowballing strategies. Researchers then contacted individuals who expressed interest in participating. Each participant only engaged in the research at one of the two timepoints (i.e., either Stage 1 or Stage 2). Recruitment continued until representation was achieved across all potential end-user groups (e.g., GPs, nurses) of the dashboard and/or recruitment strategies were exhausted. No other demographic features, such as gender or age, were considered in recruitment representation. GPs received a financial incentive in the form of monetary reimbursement.

### Data collection

Stage 1 interviews (*N* = 3) explored participants’ needs for, and access to, information; clinical decision-making processes including experience with decision support; reconciling information in RACFs and external practices; and dashboard preferences. The interview guide (Supplementary Material A) was developed and refined by the research team, including all interviewers to ensure consistency in interviewing. Interviews were conducted over the phone or via video teleconferencing software by one of three researchers (AN, LD, CM). Interview findings and dashboard design guidelines from the literature [15] informed the development of dashboard prototypes which were made of reports. In this study, the term “report” is used to characterise an electronic window specifically designed for a particular audience or a defined scope, and a dashboard comprises a compilation of reports. No demographic data on participants were collected.

In Stage 2 workshops (*N* = 20, in 8 workshops), participants viewed the dashboard prototypes and were asked to reflect on whether the dashboard was easy to understand. They suggested improvements to the content, design, and decision support functions of the dashboard. The application of conceptual and methodological frameworks by researchers to conduct workshops for the design of health informatics interventions has been ambiguous and there is insufficient evidence to suggest that one approach is superior to another [16]. However, congruent and beneficial workshop features have been identified and incorporated within the current study [16]. This includes ensuring workshops are aligned with (i) the design problem to be addressed, (ii) participant characteristics and (iii) resources available. The application of diverse formats and multisensory activities are common components used within workshops. This involves a combination of visual, auditory and tactile activities; as well as brainstorming, scenarios or vignettes as methods to elicit better interpretation of prototype application in real-world settings and understanding of participant preferences [16]. The workshop guide is included as Supplementary Material B.

**Fig. 2:**
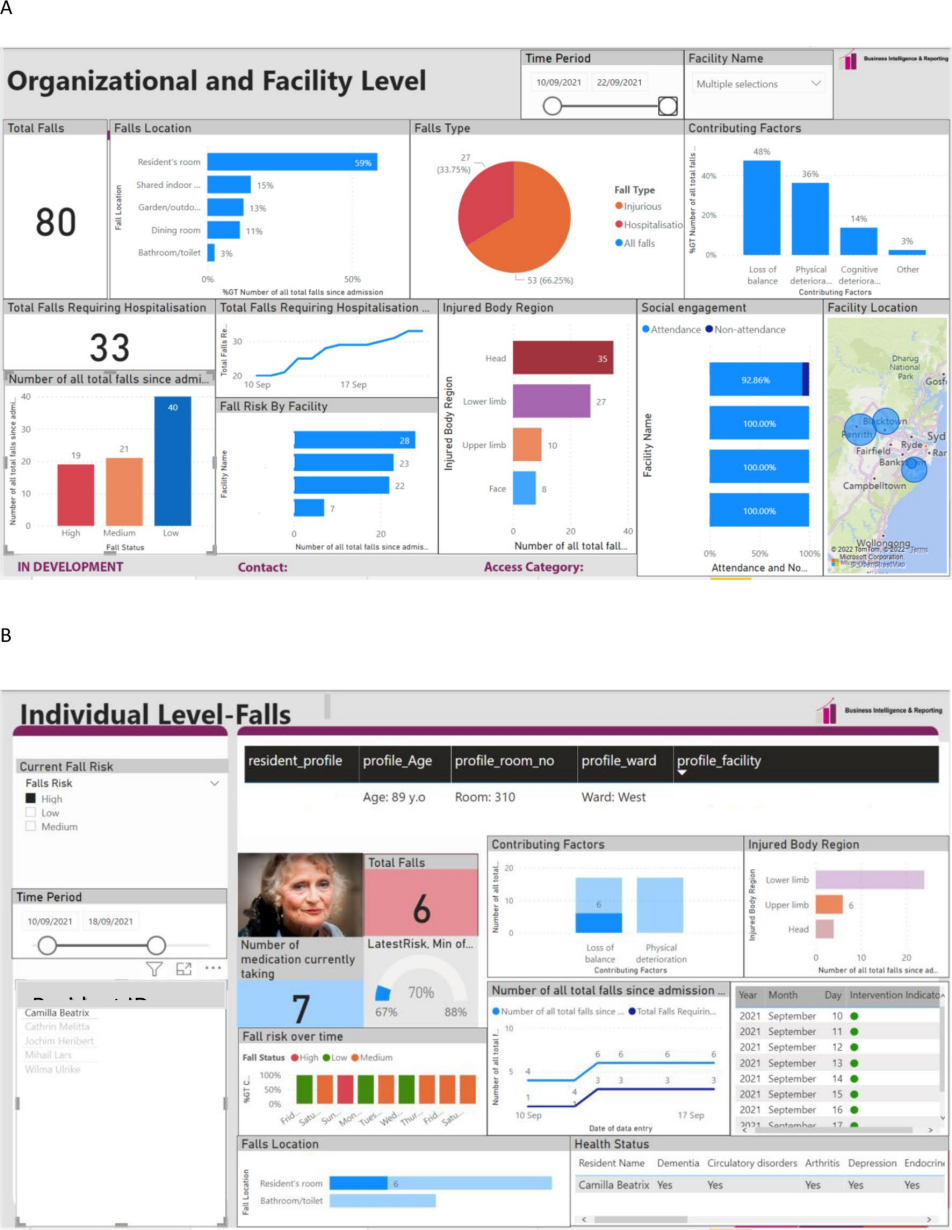
Early dashboard prototype of **A**) organisational and **B**) resident level reports, that were shared with aged care staff, GPs and geriatricians in workshops (Stage 2). Note: Names and data are fictional

Dashboard prototypes were updated between workshops in response to participant feedback via an iterative process (Fig. 1). The prototypes had two reports: individual resident (which contained data specific to each resident) and organisational (which displayed an overview of aggregate data related to resident outcomes across all provider RACFs) (Fig. 2). Workshops were conducted using either video teleconferencing software or face-to-face at RACFs, based on the participants’ preference. Workshops were conducted by 2–3 researchers per session (LD, CM, KS, SS, NW), depending on the format (online or in-person) and workshop size.

Interviewers and workshop facilitators had experience in qualitative research (LD, KS), dashboard development (SS, NW) and/or working in residential aged care settings as health professionals (LD, KS, CM). One researcher (SS), who attended all workshops, kept field notes to document participant preferences which were used to update the dashboard design between subsequent workshops. Interviews and workshops were audio recorded and transcribed verbatim using Digital & Audio Transcription Services (DAATS). The audio transcripts were manually checked by a researcher (CM) and de-identified.

### Analysis

Transcripts from Stage 1 and Stage 2 were analysed in NVivo 20 using template analysis, a type of codebook thematic analysis [15]. Template analysis is a semi-structured approach that combines elements of inductive and deductive analysis [17]. Unlike purely inductive approaches, where researchers code each transcript independently and generate codes from the data without preconceived structure, template analysis involves developing an initial coding framework based on themes identified in a subset of transcripts. This template can be informed by theoretical concepts and tailored to address specific research questions, rather than relying solely on an open-ended exploration of the data. For example, in the analysis of workshop transcripts in this study (Stage 2), the initial coding template was informed by established dashboard design principles, which emphasise content, design, and decision support as key components [15]. In template analysis, once a coding template is developed it is applied to remaining transcripts. However, the template remains flexible to accommodate new insights emerging from the data. The template analyses applied in this study followed five general steps: familiarisation, preliminary coding, theme generation, reviewing themes and writing up [18]. Interview and workshop data were analysed separately as data were collected at two timepoints for two distinct purposes.

The study required a co-design approach to make some trade-offs in the final interface of the dashboard when presenting information. For this purpose, “information prioritisation and content structuring” were undertaken utilising a measurement system [19] to inform iterations of the dashboard between workshops. During the user requirement elicitation phase, the designers identified the key information and indicators needed for the dashboard. A summary of these information needs and indicators is provided in Supplementary Material C. Following this measurement system framework, researchers were able to determine trade-offs and whether quantification of user requirements into dashboard form were relevant, complete, correct, and consistent. Moreover, conflicting user preferences such as displaying falls risk by stages (e.g., Stage 1 to Stage 4) instead of as a numerical percentage were discussed within the research team to ensure the users’ information needs were accommodated. Additionally the information requirements were organised and tailored according to different reporting levels (i.e., resident level, facility level, or organizational level). Further details on how these user requirements were organised using the measures can be found elsewhere [12].

Transcripts were analysed independently by two researchers (AN, LD [Stage 1] or IM, LD [Stage 2]). Researchers initially read transcripts to familiarise themselves with the content. Segments of data were inductively coded to identify initial themes that represented groups of codes with similar meaning. Researchers then met to consolidate codes into overarching themes by grouping codes with similar meanings and generating a coding template. All transcripts were then coded by applying and refining the coding template by one researcher (AN [Stage 1] or IM [Stage 2]). Themes were reviewed and finalised by AN and IM for Stage 1 and 2 respectively.

## Results

Stage 1 interviews were conducted with three GPs and lasted 32–48 min. Four themes were identified from the interviews: (1) Hybrid methods for accessing information, (2) Fragmented information systems and resource constraints, (3) Shared clinical decision-making in residential aged care and (4) Supporting decision-making through summarised information.

Twenty participants, including residential aged care staff (nurses and care workers), GPs and geriatricians, participated in eight Stage 2 workshops (Table 1). On average, workshops lasted 37.9 min (range 13.4–56.6 min). For residential aged care staff and GPs, workshops comprised 2–4 participants, whilst geriatrician workshops were made up of 1 participant each due to difficulties aligning participant schedules. Duration of workshops varied naturally due to maturity of the prototypes presented, number of participants in the workshop, workshop format (online or in-person), and participant availability. Themes identified included practical solutions for development of a dashboard in terms of (1) Providers’ perceptions of meaningful content (content), (2) Comprehensive information, visual communication and adaptive functionality (design), (3) Decision support for modifiable risk factors (decision support) and (4) Human-technology interaction. The final workshop coding template is provided in Supplementary Material D.

**Table 1:** Interview and workshop participant characteristics

Profession	Stage 1	Stage 2	
	Number of interviews	Number of workshops	Number of participants
Residential aged care staff		4	12
General practitioners	3	1	4
Geriatricians		3	4

### Interviews (Stage 1)

GPs were asked about several topics related to their work within RACFs including how they identified information, used it to deliver care to aged care residents, and how they would want that information presented to them in a dashboard.

### Hybrid methods for accessing information

Participants stated that they used a combination of electronic and paper-based methods to access resident information.

*“You’re working with electronic notes at the practice [and] at the [residential aged care] facility. Paper medication charts. [Observations are]*,* usually recorded electronically*,* but on a software that is incredibly cumbersome.” [GP 1]*.

Electronic methods included residents’ care plans stored within RACFs and notes the GP wrote in their own GP software. Paper formats included medication charts and handwritten notes that GPs took within RACFs either for their digital entry later into their own software or for communication with residential aged care staff, for example, a note to an RACF staff to follow-up on.

*“There is no compulsion on the GPs attending to type in the notes. So*,* you get a hybrid of notes and again*,* my writing’s pretty pathetic.” [GP 2]*.

In terms of using technology for data transfer, participants noted that fax was still frequently used in the aged care space as a method of communicating resident information between GPs and aged care staff, even though all participants agreed it was outdated.

*“We still over-rely on fax machines. I just wish we could fix that problem*,* because often faxes don’t work.” [GP 2]*.

Using contemporary electronic documentation methods like cloud technology to store information was mentioned by one participant, who said that it eliminated the need for double entry of data in RACFs and then later in their own GP software, as this process occurred automatically.

*“ [Record keeping in RACFs is] beyond not great. [Having notes] in the cloud… At least you can log in*,* see the notes*,* potentially have a look at the [observations].” [GP 1]*.

### Fragmented information systems and resource constraints

Several difficulties were described by participants regarding access to resident information. At times, the information they had on hand, both within RACFs and their own practices, was inaccurate or not up to date. This was due to a variety of reasons, predominantly that inconsistencies existed between the GP’s records and notes stored in the resident’s electronic record versus paper files within RACFs.

*“The charts at the aged care facilities are really what you go from. The charts at the practice could be quite out of date.” [GP 1]*.

Reconciling notes between the two settings was a commonly reported procedure for GPs dealing with paper and electronic methods. However, heavy workloads and slow communication between health professionals was said to often led to subsequent delays when inputting the paper notes into the electronic record.

*“Maybe you’ve had a medication review with the pharmacist*,* you might then bring that back to the practice and tidy up your records. But sometimes*,* by the time you’ve got the medication review back from the pharmacist*,* that’s three or four weeks*,* and then the charts are really out of date.” [GP 1]*.

Limited interoperability of different electronic systems between RACFs and GP practices meant that GPs needed to perform double entry of resident information into both the RACF systems and their own GP systems.

*“Quite often*,* what I do is – I have to*,* for a variety of reasons*,* type in my notes at the facility*,* go back to my [GP software] at my main base and do the identical thing … Unfortunately*,* the two systems [RACF and GP] [are] going concurrently and it’s so time consuming.” [GP 2]*.

Participants explained that information updated electronically within RACFs by people other than GPs were not included in the GPs systems. Constant data reconciliation was seen as time-consuming, meant less face-to-face time with patients and added to inaccuracies as this process was rarely completed for all changes to a resident’s file. GPs explained that this meant information was missing.

*“We’ve got doctors [in RACF] who look after 30 or 40 patients… And when they go [to the facility] … they might see 12 or 14 of them. They’re not going to enter the whole data. First of all*,* [they record it] on the paper… Then they’ve got to enter it into the software of the aged care facility. They’ve got to come back to the practice*,* remember everything*,* and enter everything again.” [GP 1]*.

*“I’m not a great lover of spending hours and hours on the screen and the whole idea of transferring stuff back and forward*,* cutting and pasting… all of that means more time at the screen and less time face to face with patients.” [GP 3]*.

My Health Record, a national online suppository of health information, was suggested as a solution to double entry; however, GPs stated that there was little information stored within My Health Record currently.

*“There’s no cloud storage. And it’s got to happen. I’ve been so involved over the years with My Health Record right from its inception. But it’s just not working*,* I’m still using it intensely*,* but I can’t see any action around me.” [GP 2]*.

There was mutual agreement that there are often no permanent GPs on site at RACFs; most have scheduled visit block times or consult via phone call. This meant that gathering and sharing information about a resident from the GP perspective was challenging, especially when attempting to collect accurate and timely medical information on a resident from a registered nurse or relay relevant messages about the resident to the most appropriate staff members.

*“So*,* you decide – I may as well go in [to a RACF]*,* because this is not a reliable history now… Then the first thing that’s a problem is finding a nurse … Either they don’t know or aren’t particularly interested… Then eventually you find a nurse and they may or may not have the information.” [GP 1]*.

Similarly, participants said that when they tried to look through a resident’s file stored within RACFs, it was at times difficult to understand why certain decisions were made due to the system logic (e.g., system requires many clicks to reach relevant information) or the person who entered the information did not provide sufficient supporting information to explain their decision-making, for example, indication for a medication being added or removed.

*“ [Another clinician] might have changed some of the medications and it’s really difficult to work my way through the logic as to why things have changed*,* let alone getting correct information from nurses who are often part time … There’s significant problems in aged care to do with getting quality information from other health professionals at various levels.” [GP 2]*.

*“I put stuff in the notes… Very rarely does anyone read each other’s notes*,* because they don’t have time. The whole culture is that you’ve got to note everything*,* but unfortunately the time taken on noting everything doesn’t give most [registered nurses] any time to read the stuff” [GP 3]*.

### Shared clinical decision-making in residential aged care

There was agreement that the most common decisions made by GPs within RACFs were about medications, for example, changing doses, adding medications, and deprescribing. Other clinical decisions GPs made referred to alternative treatments for conditions such as constipation, sleeping difficulties, skin tears, behavioural issues and determining falls risks. Participants indicated that GPs were also instrumental in deciding whether a resident should be sent to hospital. Participants acknowledged that clinical decisions made within RACFs were shared decisions and included input from families, RACF nurses, pharmacists, allied health (e.g., physiotherapists, podiatrists, dietitians) and specialists.

*“We have lots of family case conferences where we nut out problems. There’s the pharmacists that attended the facilities… There’s the various different allied health people at all different levels. There’s podiatrists*,* physiotherapists*,* dieticians and at some facilities*,* we might have chaplains. So*,* it’s a teamwork situation.” [GP 2]*.

### Supporting decision-making through summarised information

Statements from participants demonstrated that decision support is rarely used in residential aged care. However in their own practices they used a variety of decision support tools such as accessing therapeutic guidelines, setting reminders, creating to-do lists, and using assessment scales, to support their clinical decision-making.

*“The only way I can remember when that specific patient needs to be recategorised*,* is I’ve got a to do list that I put months ahead. I’ve got to add it into my reminders box in [GP software] … There are all these little things that are lacking in a system at the aged care facility that I’ve got to add into my own hardware or some process … I just can’t rely on the facility or the nurses to remind me*,* because they often get it wrong.” [GP 2]*.

When asked about a design of the dashboard, participants said that having a summary of recent health status changes that a resident experienced would support them in their decision-making, as it would allow them to see an overview of a resident who they may have not seen in a while.

*“I would initially start using the most basic available information. Just an ability to =[to view]*,* their [observations] There might be an arrow that points up or points down …. The number might appear in green if it’s within a set range that the doctors set. It might appear in red if it’s outside that.” [GP 1]*.

### Workshops (Stage 2)

Practical solutions for the development of a dashboard were raised by workshop participants which reflected three themes: providers’ perceptions of meaningful content (i.e., information included or missing on the dashboard), design: comprehensive information, visual communication and adaptive functionality (e.g., colour, graph types, positioning), and decision support for modifiable risk factors. A summary table of practical solutions is provided in Table 2. An additional theme was identified, “human technology interactions”, which had three subthemes: mistrust, real world applications, and barriers to dashboard use.

**Table 2:** Summary of practical solutions for the falls risk dashboard

Content	Design	Decision support
Not needed: • Older falls history • International classification of patient safety results Needed: • Medication-related data • Mobility status Mixed opinions: • Resident medical history	• No/minimal alerts • Filtering functions • Drill down functions to link reports • Simple line and bar graphs • Consistent colours (in aged care provider theme) • Labels • View (spacing, organisation, simpler plots etc.) • Benchmarking against other facilities	• Timely decision support • Identify modifiable risk factors • Evidenced-based support

### Providers’ perceptions of meaningful content

When asked about the dashboard prototype, participants discussed what content they believed was missing from the prototypes and what sections were classified as not useful. Missing content largely included medication data as patient medication was not included in early versions of the dashboard. This was later added along with identifiers for falls risk enhancing drugs. Content reported by participants to be less relevant included older falls history, type of fall and cause of falls (entered by staff in fall incident reports). The rationale for this included the ambiguous way this data was coded and low variance (i.e., the majority have “poor balance”) which did not give a meaningful summary of a resident’s condition.

*“It doesn’t have the medications though*,* which for us would probably be one of the more useful things” [Geriatrician 2*,* Geriatrician workshop 2].*

Opinions differed regarding the level of detail that should be included in the data displayed in the prototype. For some participants, the necessary data only included fall risk enhancing drugs or the number of drugs, other health professionals saw utility in including a complete list. Similarly, it was seen that some health professionals wanted to include a full list of resident conditions, however, others mentioned possible redundancy, that this information would likely be well known by users. It was also mentioned in discussions with some participants, the inclusion of a tailored list of medications which were directly relevant to falls.

*“When I look at the comorbidities*,* they’re not terribly helpful*,* usually I know them already.” [GP2*,* GP workshop 1]*.

*“Of course*,* when you have issues like arthritis*,* cataract*,* any vision problems*,* they are equally contributing towards the comorbidity [falls].” [Residential staff member 2*,* Residential staff workshop 2]*.

There was consensus on some aspects of the dashboard content. For example, participants consistently requested the most recent mobility status to be available on the falls dashboard. There was also agreement on the predictive component of the dashboard, participants stated that predicting falls risk was useful. However, they were uncertain whether a percentage was the best measure of risk and believed that the factors driving the falls risk needed to be highlighted.

*“Obviously*,* we don’t want [falls risk] to be found because the staff look and say*,* ‘Their falls risk was at 58 per cent yesterday*,* and it’s now 65 per cent*,* can you come and review them?’ It needs to be sensible as well*,* that we’re not just looking at numbers on a dashboard and then calling the panic button thing.” [Geriatrician 1*,* Geriatrician workshop 1]*.

*“[Falls risk] is a good idea*,* as long as the source data is clear” [Residential staff member 2*,* Residential staff workshop 2]*.

### Design: comprehensive information, visual communication and adaptive functionality

There was a shared perspective that the dashboard needed to be strongly visual with a focus on graphs. Participants said that they preferred dashboard prototypes with line or bar graphs and consistent colours. The graphs also needed to be well labelled as not all terms were familiar to clinicians (e.g., fall risk increasing drugs [FRIDs]) and values were unclear.

*“It’s not obvious on the chart that this is the risk for the next month.” [Geriatrician 5*,* Geriatrician workshop 3]*.

When given the option between a simple view (i.e., fewer graphs and text) or a more complex view, participants often opted for the latter as they liked being able to view the maximum amount of information possible without needing to open another window. Suggestions on how to purposefully design the available screen space to achieve this included reducing or removing meta data (contextual data on facility and resident details such as name and age) and filtering menus and buttons. Some participants wanted drill down and filtering functions so they could still explore data as they chose, but they admitted in their everyday practice it would be unlikely that they would have the time to undertake such action. Not all participants believed that drill down functions were needed.

*“I’m sick of clicking boxes and trying to get to another screen and another screen and another screen.” [GP 2*,* GP workshop 1]*.

*“If for example*,* we could click five and it would just pop up a list of the meds*,* that would be useful*,* rather than me having to find the nurse to unlock the cupboard to get the folder of med charts out to – or to look at my list on my software which might not be up to date.” [GP 1*,* GP workshop 1]*.

Earlier iterations of dashboard prototype designs placed emphasis on predictive falls risk scores. There was a focus on optimised utility of the dashboard screens, to minimise the need for participants to move between pages. During the workshops, it was expressed that emphasis should be placed on fall history, modifiable risk factors and targeted falls prevention interventions.

*“There’s a lot of screen taken up by that left column… the facility and ward*,* resident name. All that doesn’t really need to be there. We just select the resident and then it’s up. The big box that says “high”*,* it’s wasted space really. We can see it’s high – it’s got the high little graph next to it… You could use the screen space for other things to make it more interesting.” [GP 2*,* GP workshop 1]*.

In relation to the falls history presentation, participants explained that the number of falls could be more meaningfully benchmarked against other participating facilities by using rate comparisons rather than number of falls per resident. Participants expressed that they strongly disliked the use of alerts—pop-up functions notifying clinicians of a change—instead preferring to have their attention drawn to any changes by generating a summary page or a change in the colour on the resident’s profile.

*“If you could be alerted if someone’s had a fall in the last 24 hours or if something’s changed*,* that would probably be ideal.” [Residential staff member 1*,* Residential staff workshop 1]*.

### Decision support for modifiable risk factors

No decision support was presented in the initial prototype of the dashboard. However, participants described that they expected the dashboard to highlight modifiable risk factors at various levels as part of the decision support. Modifiable risk factors are reasons that increase the risk of a resident having a fall but have an action that can be conducted to minimise the risk of falling. An example of a modifiable risk factor included participants expressing interest in viewing medication-related indicators that may contribute to falls risk.

*“Nothing on medications in terms of contributing factors*,* so polypharmacy’s a contributor and centrally acting medications?” [Geriatrician 2*,* Geriatrician workshop 2].*

Participants also highlighted the need for guidance on appropriate next steps that they could conduct when a falls risk is identified, including specific evidence-based interventions such as preventive measures (e.g., hip protectors, bed rails, crash mats, lowering the bed), environmental modifications (e.g., decluttering the room), and medication management guidelines.

*“You probably want a little window that picks out the things that you believe can be changed.” [Geriatrician 1*,* Geriatrician workshop 3]*.

“*Staff need to be guided to prevent Mrs Smith from falling. Not to know that she’s got a 91% chance of falling today*,* but actually here is what I need to do to stop her from falling.*” *[Geriatrician 2*,* Geriatrician workshop 2]*.

### Human-technology interaction

Through the workshops it was recognised that the feasibility of the dashboard depended on more than a streamlined interface. Three subthemes were identified: mistrust, real world applications, and barriers.

### Mistrust of data accuracy

Many participants—GPs and geriatricians in particular—expressed mistrust with the source of the data and how data is represented. They reported that without this trust and transparency, they would likely disregard the information as they did not believe it was accurate. Possible solutions to mistrust issues proposed by participants included clear and consistent labelling on the dashboard so that healthcare professionals could identify where in the EHRs the data came from. Participants were also wary of the need to enter additional data into the dashboard for it to work appropriately, even though the dashboard only uses routinely collected data inside EHRs.

*“We always look at it from the time perspective in the [RACF]*,* how time poor [registered nurses] are … Do they have to be feeding data somewhere by the [RACF]*,* or it works directly with our clinical system?” [Residential staff member 2*,* Residential staff workshop 2]*.

### Real world application of the dashboard

Some participants expressed that the dashboard would be beneficial to resident care and as well as their workflows. Real word applications suggested by participants included very pragmatic approaches as to how the information from the dashboard may be used. For RACF staff, this often included a list of new referrals to other professions.

*“Residential staff member 4: Physio to review his mobility…*

*Residential staff member 5: Need some blood test done; he should be on vitamin D. Have a look into other risk. More supervision [of the resident] required; maybe more assistance required. What else*,* and the look into the lifestyle*,* the time of the day*,* what time he’s falling.” [Resident staff workshop 3]*.

Others raised concerns about how the information would be used in practice, either believing it would not impact practice at all or have the potential to cause harm.

*“So*,* if I come into work*,* I’m the [registered nurse] looking after Mrs Smith*,* I see today’s falls risk is 91%*,* I would be paranoid that Mrs Smith is about to fall today. I might get her to sit down and do nothing all day*,* so she doesn’t fall.” [Geriatrician 1*,* Geriatrician workshop 2]*.

### Infrastructure barriers to dashboard development, use and implementation

Infrastructure barriers to dashboard use were identified by participants during interview/workshops. These included poor internet connectivity, limited number of computers available, inability to train all staff (e.g., nurses from external staffing agency) and lack of time to read notes and handover. Also mentioned were components of documentation that may be an issue with dashboard implementation such as the presence of paper notes inside a resident room, with mobility status and fall risk that may not be updated or consistent with EHRs and therefore not fully integrated into practice.

*“Residential staff member 4: We could take the iPads with us to the handover*,* but it might extend handover a bit too much …*.

*Residential staff member 5: Someone will be monitoring [registered nurses] and care coordinators and the care manager will be monitoring.*

*Residential staff member 4: We’ll encourage the care staff to look at it [dashboard]*,* but they probably won’t.”* [*Residential staff workshop 2]*.

## Discussion

This study aimed to understand informational needs of healthcare professionals working in RACFs and their preferences for a digital falls dashboard to support their delivery of care. In initial interviews which scoped existing information transfers, GPs reported inaccurate resident data and difficulties in reconciling information across care settings due to the hybrid nature of aged care (i.e., paper based and electronic) and that majority of clinical decisions were made about medications, with minimal use of decision support. These findings informed prototypes of a falls dashboard. The design of the dashboard prototypes, including the content and decision support functionality, which were refined in workshops. Workshop participants wanted medication and mobility data to be displayed in the dashboard, as well as benchmarking, with functionality to filter and drill down results, and provision of evidence-based decision support, but with minimal alerts.

**Fig. 3:**
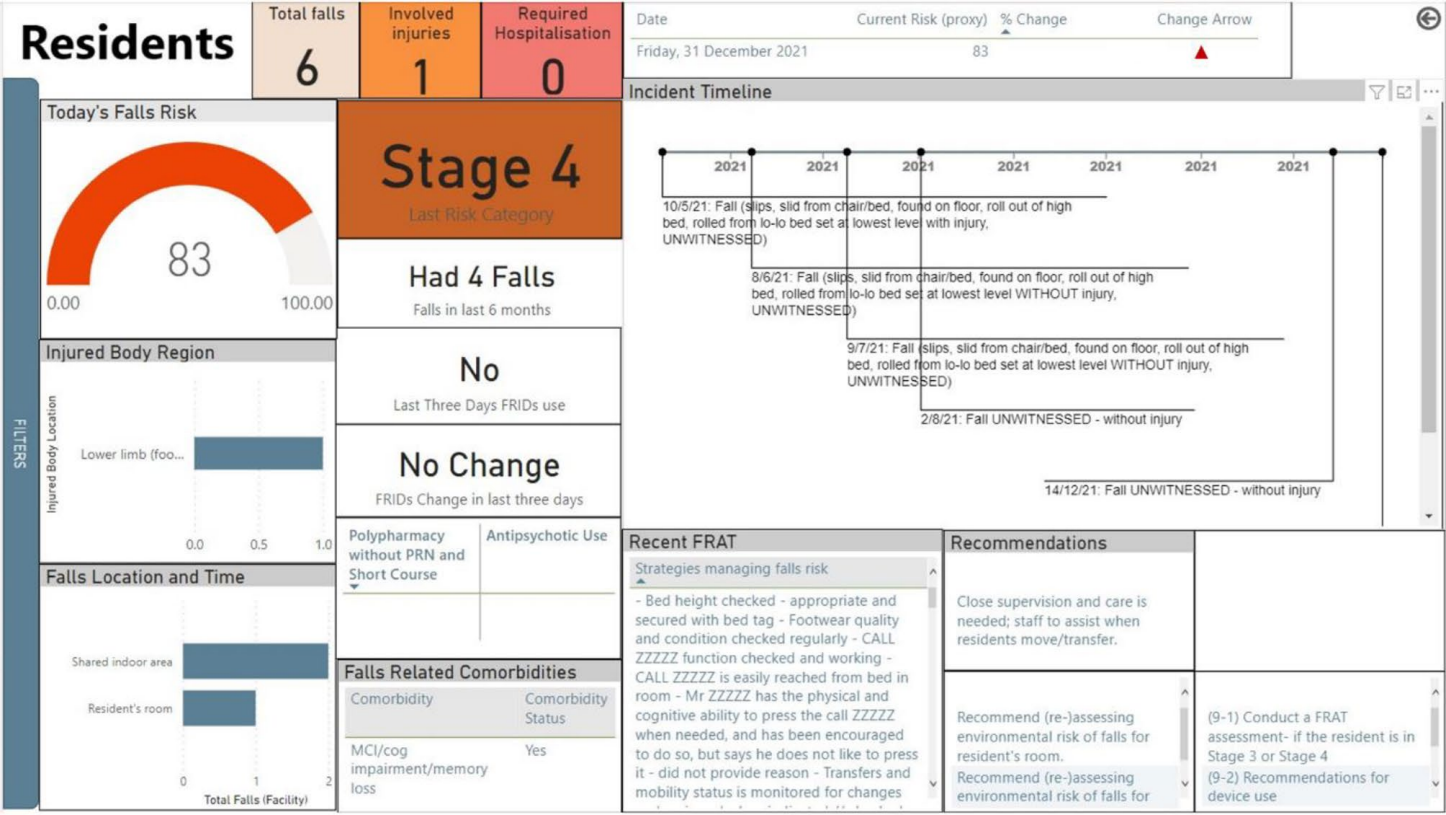
Later iteration of resident level report in the dashboard after Stage 2 workshops. Note: Names and data are fictional

A co- design approach was adopted to balance the needs of different healthcare professionals within residential aged care. To meet end-user needs, the dashboard became more dynamic throughout the co-design process, with the inclusion of interactive features such as expandable reports, filtering and drill down functions and more targeted content, focusing on falls risk factors and prevention interventions (Fig. 3). Although two reports, for an organisational level and a resident level, were designed and presented initially, this number increased following workshops to also include reports to depict the facility level information, resident level information (such as medicine in detail) and provide recommendations to staff (i.e., on resident level report and as well as in a separate report to enhance the staff education).

The design of the original dashboard prototype aligned with the available literature on dashboard design: traffic light colouring to signal performance, visual display, simple graphs [20, 21] and peer comparisons [22, 23]. While the most recent dashboard design still had many of these features, it was modified based on participants’ feedback during the co-design methodologies. A feature that participants preferred, but that deviated from evidence [20, 24, 25], was more crowded dashboard displays. Evidence to guide dashboard design is limited and where available, lacks specificity. For example, previous researchers have recommended that dashboards should not be too crowded [20, 26], but it is unclear what represents “too crowded”. Dashboard design is likely highly subjective, dependent on the context, and characteristics lie on a spectrum. While more evidence on dashboard design and use in healthcare is needed, specific design features (e.g., how much or how little of specific features) will always require end-user input to meet user needs in their context of use.

Participants in this study were specifically asked about their falls management and how a dashboard could support these processes. From participant feedback, a rule-based recommendation system was implemented within the dashboard to support decision-making process around falls prevention. Such strategies would not only minimise double entry by reconciling paper and electronic forms of data but allow for accurate and timely information sharing, fostering a sense of trust in the data and freeing up time for healthcare providers to provide quality care to residents. These strategies, amongst other benefits of data integration and analytics, such as overcoming the skills gap in data interpretation, assisting proactive care, and service planning and improvement, have been cited in the literature on electronic decision support within healthcare [27, 28]. Our study highlights that this sentiment is shared amongst GPs, geriatricians and staff working in RACFs, which have been slower to uptake new technologies.

The dashboard prototypes were developed using participant data extracted from the aged care provider into Microsoft Excel spreadsheets. During the development process, the aged care provider changed both its incident reporting system and daily medication management system, which were initially used for data extraction. To ensure the dashboard remains sustainable despite these underlying system changes, a data reservoir was implemented between the data sources and the dashboard. This intermediate layer will play a critical role in ensuring the dashboard’s long-term sustainability and scalability across a broader range of aged care environments. These technical challenges, along with the strategies adopted to address them and future directions, have been previously discussed [12].

An overarching theme of distrust towards resident information presented to RACF staff emerged from both interviews and workshops which could represent a significant barrier towards the integration of a digital falls dashboard. In interviews, GPs stated that at times, resident information was out of date, changed by other healthcare providers, or did not include adequate detail to support clinical decision-making. For example, other healthcare providers might not provide reasons for making changes to information, or they might not give a rationale for a clinical decision, such as why a medication was prescribed or ceased. Additionally, compounding system and process barriers reported by workshop participants included limited time to read resident notes, short handovers, and the combined use of paper notes that are inconsistent with information in EHRs. These findings are similar to previous research conducted with physicians and nurses regarding barriers to use of electronic decision support tools (e.g., e-prescribing). This includes missing structured diagnosis documentation and problematic false alerts based on outdated medication information and paper-based charts [29, 30]. However, participants suggested possible solutions to overcome these barriers focused on minimising the amount of data that exist in silos. Some GP participants suggested utilising the advantages of existing technology, for example, the automaticity and interoperability of the cloud system or an electronic information repository such as My Health Record. These recommendations are consistent with the advantages provided by a clinical dashboard (i.e., interoperability and automaticity [23]), further supporting its potential adoption into workplace practices for multiple levels of aged care staff.

### Future research

Choosing one design feature over another can create trade-offs in application and use. For example, increasing the space on one page may push the dashboard into multiple pages or the ability to filter and query information may remove the number of pages. Dashboard end-user reports (i.e. person, facility, organisational) that targeted multiple layers of decision-making processes regarding falls prevention was identified through workshop insights. These design choices have practical implications; thus, they require end-users to have better training to use the dashboard or important information may be missed. As the dashboard continues to develop based on feedback during implementation, it is important that the design remains not only user-centred, but goal-orientated for the target audience. A potential solution to accommodate contrasting perspectives could be adopting a customisable design. Dashboard design in aged care is in its infancy and regulations surrounding inclusion of obligatory data necessary for resident safety within customisable dashboards has not yet been investigated. Thus, consultation with experts in dashboard design and development, clinicians, aged care professionals and key regulatory bodies may be warranted in future.

Some evidence suggests that dashboards may be more effective in changing end-user behaviour when implemented with additional interventions, for example, team huddles and staff training [31]. However, this evidence is not specific to falls outcomes or aged care settings. Other systems, such as Veteran Affairs in the USA, have attempted to understanding barriers and facilitators, used implementation theory, and worked closely with end-users to try to facilitate successful dashboard implementation [32]. Future research on aged care dashboard design should aim to involve larger and more diverse samples representing the full spectrum of stakeholders, including aged care residents, family members, healthcare professionals, and IT personnel. This broader engagement is essential to ensure that the tool reflects the needs, preferences, and practical requirements of all end-users and contributors to its development and implementation. For this specific dashboard, our research team has also explored the perspectives of aged care consumers and their families regarding the design and functionality of digital dashboard [33]. That study highlighted the value consumers placed on features such as large text, intuitive symbols, and the ability to tailor the interface to individual needs. Building on this, the present study shifts focus to aged care professionals, examining their perspectives on dashboard design and functionality to ensure that future tools meet the needs of both end-users and service providers. The next phases of our project involve finalising the dashboard design, implementing it into practice at a RACF, and subsequent evaluation of its usability, feasibility and clinical effectiveness using a randomised clinical trial [4]. Key areas of investigation will include the impact on care workflows, communication, day-to-day operations, and care outcomes, with the goal of ensuring that the dashboard is not only effective, but also sustainable and scalable across varied aged care contexts. This is important to demonstrate not only the usability of the dashboard, but to ensure the dashboard is relevant to the intended users and importantly complementary to their current work processes.

### Strengths and limitations

This study contributes to the limited literature on digital technology interventions in RACFs, by employing co-design methodology to design a falls dashboard with potential end-users. Our findings align with previous research in which dashboard end-users often have different roles with unique information needs [34]. This was emphasised during user requirement elicitation phase in which some participants’ preferences for dashboard features contrasted with what others wanted (e.g., some participants wanted drill down functions while others did not). A key strength of the study was its methodology in synthesising contrasting preferences. Combining the dashboard features identified in interviews through information prioritisation and content restructuring into multiple prototypes across end-user workshops allowed researchers to effectively balance trade-offs with customisation. Conflicting user preferences were reconciled by creating dashboard prototypes that were tailored to different reporting levels (i.e., resident level, facility level, or organizational level) which assist with eliminating implementation issues regarding acceptance and applicability within next phases of the project [35]. 

However, this study also had several limitations. First, a larger sample of participants (*n* = 50) was the original recruitment aim, however, after months of exhaustive recruitment strategies, participation rates remained low. Low participation in this research effort may be due to staff burnout as aged care providers were under immense pressure during the COVID-19 pandemic, when this project was conducted. As a solution, development of future digital interventions in residential and healthcare settings could employ predictive AI tools to assist in co-designing interventions [36, 37]. Such tools are used to extract key constructs to inform intervention development and continual monitoring of intervention impacts despite smaller sample sizes. Second, the sample collected may not be representative of all the professions who will use the dashboard. This is particularly pertinent considering the small number of participants in interviews. This may impact on the generalisability of the findings, however interview findings on information needs and dashboard preferences were further discussed with more and with a wider range of healthcare professionals in the workshops, for validity. Participants identified that registered nurses and allied health professionals were the most likely professions to use the dashboard; however, many RACF staff participants were personal support workers and managers. Other demographic information which could have impacted perspectives such as age and experience with EHR/IT systems, was not collected and is another limitation of this study. In future phases of the project, further efforts will be made to work with nurses and allied health to tailor design and implementation of the dashboard to their needs.

## Conclusion

This research involved close collaboration with proposed end-users of a digital falls dashboard being designed for RACFS. Healthcare professional participants discussed their current information seeking behaviours, which were largely uncoordinated and required improvement. Participants perceived that information gathering could be supported using a digital dashboard and provided preferences for the presentation and functionality of such a dashboard. Provision of relevant information through text and visual graphics and tailorable presentation of information were suggested strategies to improve the use of a falls dashboard currently being designed. This is particularly important in aged care where digital health is still in its infancy, and thus there is limited evidence regarding what digital support is required in this setting. Thus, to foster long-term adoption and use of digital dashboards in practice, it is fundamental to work with relevant stakeholders to understand work practices and needs, and to inform the development of trustworthy and sustainable digital solutions for tasks identified as requiring improved efficiency or accuracy.

## Supplementary Information

Below is the link to the electronic supplementary material.

Supplementary Material 1

Supplementary Material 2

Supplementary Material 3

Supplementary Material 4

## Data Availability

The datasets used and/or analysed during the current study available from the corresponding author on reasonable request.
