# Supplementary material for: Simple but complex: aged care healthcare professionals’ perspectives on the design of a digital falls dashboard
Source: BMC Med Inform Decis Mak. 2025 Sep 29;25:347. doi: 10.1186/s12911-025-03135-z (PMC12482382; doi:10.1186/s12911-025-03135-z)
Supplement: Supplementary file 1 — Supplementary Material 1 [file 12911_2025_3135_MOESM1_ESM.docx]

# GPs – Interview guide

## Access in RACFs

1. Please describe what information you access in RACFs.
2. How do you access this information?
3. Where is this information stored in the RACF? (before and after use)
4. Are there any difficulties you have accessing resident information, either currently or in the past?

## Decision making

1. Besides yourself, who is involved in making clinical decisions of residents?
2. What are the most common types of clinical decision you make?
3. Is there any information that you do not currently have access to that you think would help you make decisions about residents’ care?
4. What are your experiences with using decision support in making clinical decisions?

## Access outside RACFs

1. Do you make decisions about residents when you are off-site? Please describe how this process works.
2. How do you access resident information when you are off-site?
3. How accurate is this off-site information?
4. How do you reconcile your records between RACFs and your practice?

## Home care

1. Can you tell me about your interactions with home care clients and providers?
2. How do you access information about home care clients?
3. What opportunities do you think there are for extending your services into the home care space?
4. How do you think partnerships between GPs and home care providers could be improved to enhance care for clients?

## Electronic data

1. How comfortable are you with using technology?
2. Do you use electronic health records for residents? Why/not?
3. Do you have any other comments or suggestions about this research?
