# Supplementary material for: Simple but complex: aged care healthcare professionals’ perspectives on the design of a digital falls dashboard
Source: BMC Med Inform Decis Mak. 2025 Sep 29;25:347. doi: 10.1186/s12911-025-03135-z (PMC12482382; doi:10.1186/s12911-025-03135-z)
Supplement: Supplementary file 2 — Supplementary Material 2 [file 12911_2025_3135_MOESM2_ESM.docx]

# Aged care staff workshop guide

**Information seeking**

1. Please provide some examples of situations when you would look for information about clients/residents.

**Information accessibility**

1. What are the common types of information you are accessing?
2. Where do you get this information from?
3. Is there some information that is difficult to access or understand?

**Decision-making**

1. What are the most common types of decisions you make about providing care to clients/residents?
   1. What do you prioritise highly and does this change throughout the day/why?
2. Who else is involved in this decision making process?
   1. How are they involved/how do you communicate information
3. What is your experience with decision support in making clinical decisions
4. In situations where information that you need is not available, what back-ups or workarounds do you use to make decisions?

**Dashboard preferences**

1. The dashboard will display clients’ care and health information. What information would you like the dashboard to display?
2. What forms would like you the dashboard to link to?
3. Now I will ask you a few questions about how you want the dashboard to look. Please explain how you feel about:
   1. The use of colours to show areas clients are doing well or not well in?
   2. The amount of text that should be used?
   3. The use of pictures or symbols?
   4. The use of graphs, for example to see changes over time?
   5. The amount of information the dashboard should show?
   6. In-built decision support features?
