# Supplementary material for: Simple but complex: aged care healthcare professionals’ perspectives on the design of a digital falls dashboard
Source: BMC Med Inform Decis Mak. 2025 Sep 29;25:347. doi: 10.1186/s12911-025-03135-z (PMC12482382; doi:10.1186/s12911-025-03135-z)
Supplement: Supplementary file 4 — Supplementary Material 4 [file 12911_2025_3135_MOESM4_ESM.docx]

## Supplementary material C

**Table1. Workshop coding template**

| **Theme** | **Subtheme** | **Description** |
| --- | --- | --- |
| ***Providers’ perceptions of meaningful content*** | | |
|  | Helpful content | Information in the dashboard prototype that participants reported as useful to their practice or commented on positively. |
|  | Missing content | Information participants identified as necessary for their practice but noted was not currently available in the dashboard prototype. |
|  | Unhelpful content | Information included in the dashboard that participants felt did not add value to their practice or decision-making. |
|  | Unclear content | Information that participants found difficult to understand, such as unclear graphs or the use of unexplained acronyms. |
| ***Design: Comprehensive information, visual communication and adaptive functionality*** | | |
|  | Filtering | Participant feedback on filtering options available in the dashboard prototypes, such as filtering by resident, date or facility. |
|  | Graphs | Participant feedback on graph types, outcome measures represented in graphs (e.g., rate vs. number of falls), labels, and graph formatting. |
|  | Layout | Participant feedback on the placement, quantity, and design of dashboard features, including aspects like shape, size, colour, and font. This subtheme occasionally overlapped with "Unhelpful content" when layout concerns were discussed in conjunction with content issues. |
|  | Alerts and visual representation of dynamic features | Participant feedback on prompts and visual cues in the dashboard (e.g., email reminders, colour/icon changes indicating resident status updates). |
|  | Drill down/click through | Participant feedback on interactive drill down features, such as the ability to click on a graph to access more detailed information or navigate to another screen with additional detail. |
| ***Decision support*** | |  |
|  | Modifiable risk factors | Participants expressed a preference for decision support tools that highlight modifiable fall risk factors (e.g., high sedative load) rather than non-modifiable ones (e.g., age), to ensure the feedback is actionable. |
|  | Design with clinicians and evidence | Participants highlighted the importance of aligning decision support with evidence and involving clinicians in its design. |
| ***Human technology interaction*** | |  |
|  | Mistrust | Participants discussed concerns about data accuracy, including errors in data entry (e.g., fall reporting) and unclear presentation (e.g., combined fall risk measures without explanation of calculation). |
|  | Real world application | Participants shared how they envisioned using the dashboard in practice and expressed, in some cases, doubts about its usefulness in clinical practice. |
|  | Barriers | Participants discussed implementation barriers such as limited number of computers available at aged care facilities and time poor staff. |
